# An evolutionary perspective on kin care directed up the generations

**DOI:** 10.1038/s41598-021-93652-4

**Published:** 2021-07-08

**Authors:** Megan Arnot, Ruth Mace

**Affiliations:** Department of Anthropology, University College London, London, WC1E 6BT UK

**Keywords:** Biological anthropology, Behavioural ecology

## Abstract

Within evolutionary sciences, care towards younger kin is well understood from an inclusive fitness framework, but why adults would care for older relatives has been less well researched. One existing model has argued that care directed towards elderly parents might be adaptive because of their benefits as carers themselves, with their help freeing up the middle generations’ energy which can then be invested into direct reproduction. However, in this model, elder care is more beneficial to fitness if the carer is fecund. To offer an initial test of this hypothesis, we look at caring behaviour relative to fecundity status in a contemporary dataset from the United Kingdom. If elder care is contingent on possible direct fitness benefits, we would expect women who are still menstruating to care more for their parents than women who can no longer reproduce. Based on this, we also predict that women who are physiologically post-reproductive would invest more in their grandchildren, through whom they can increase their inclusive fitness. After controlling for age and other relevant factors, we find that women who are still menstruating spend more time caring for their parents than those who are not, and the reverse is true when looking at time spent caring for grandchildren. These findings demonstrate that potential inclusive fitness outcomes influence how women allocate care up and down the generations.

## Introduction

Kin care is ubiquitous in human cultures, with cooperative breeding being a key component of our success as a species^1,2^. While kin structures in many contemporary societies have shifted dramatically in recent years, there are still societal expectations in place for kin to assist in allocaring. Usually investment flows through families down the generations: younger kin are helped by older kin^3–5^; and the amount of allocare received is generally predicted by degree of relatedness and the reproductive value of the recipient, so that any costs incurred by helping will be offset by the possible inclusive fitness benefits^6–8^. However, it is common to observe younger individuals caring for older family members such as parents and grandparents. Cultural norms promoting elder care are widespread^9–11^, including in hunter-gatherer populations^12,13^, and such behaviour is encoded in many religions and moral codes, with these injunctive norms being further institutionalised in some countries where there are laws making filial care (care from children to parents) a legal requirement^14,15^. Despite the prevalence of elder care, it is poorly understood and seldom empirically researched from an evolutionary perspective.

Much of the evolutionary research looking at kin care has focussed on care flowing down the generations. As a result, there is an ever-growing body of empirical research demonstrating the inclusive fitness benefits associated with care directed from older family members to younger ones^2,16–20^, with such indirect fitness gains thought to be responsible for the maintenance of the female post-reproductive lifespan^2^. While the principles of kin selection can explain why people invest heavily in younger kin who are reproductive, why adults would invest in their older relatives is less clear from a fitness perspective. Older women are likely to be post-menopausal and therefore unable to directly reproduce, making the possible indirect fitness gains minimal; and fathers of adults may physiologically be able to reproduce in old age, but their fertility will be constrained by mate availability, as it is likely these older men will be partnered with women of a similar age who are post-menopausal making reproduction unlikely^21^. Despite the apparent limited inclusive fitness gains to be had from the behaviour, caring for elderly parents is a cross-cultural norm.

**Figure 1:**
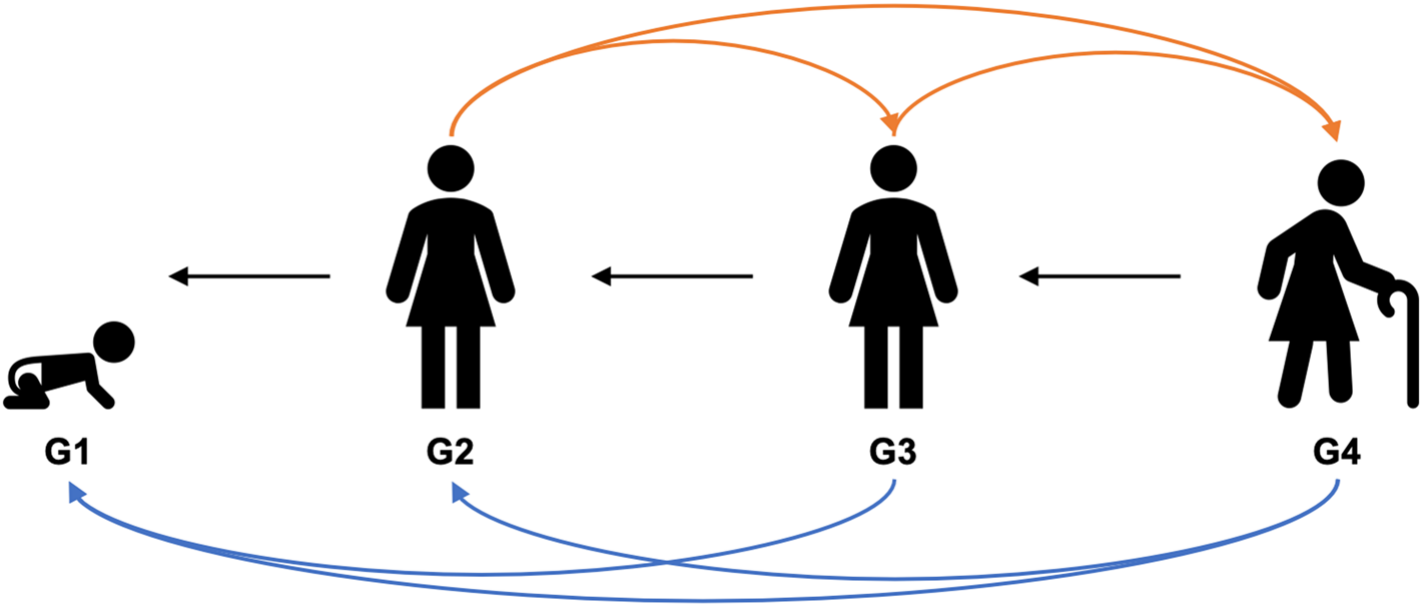
Graphical description of the model proposed by Garay et al. suggesting that elder care would have evolved because of their benefits as carers themselves. Here, care from G3 to G4 is thought to be adaptive if G4 invests more in the younger generations (e.g. G1, G2) which frees up G3s energy that can be allocated to increasing direct fitness. Upwards intergenerational care is represented by orange arrows, downwards intergenerational care by blue arrows, and black arrows indicate parental care.

At present, we are only aware of one model attempting to address elder care from a fitness maximising framework^22^. Here, in a demographic model it is proposed that upwards intergenerational care would be selected for due to the possible reciprocal benefits of the behaviour. Graphically described in Fig. 1, this model proposes that if an adult (e.g. G3 in Fig. 1) directs care towards an older adult (e.g. G4 in Fig. 1), it will make the latter group more able to offer downward intergenerational care, such as to grandchildren or great-grandchildren (e.g. G4 to G1 in Fig. 1). This additional instrumental help from elderly family members (i.e. in the form of additional childcare) decreases the burden of parental care for the middle generation adults, which frees up some of their energy that can be invested into other activities such as foraging (which they would do with higher efficacy than older kin) or reproduction^23^. Therefore, care towards the elderly would be selected for due to their benefits as carers themselves. Though the elderly would already have an incentive to invest in younger kin as it is the only route to increase their inclusive fitness, it is thought that receiving care from adult relatives makes the elderly *more able* to offer direct care towards younger relatives. This additional support from the elderly then allows fertile adults to allocate their energy into other activities, such as increasing their direct fitness through reproduction (which would not be possible for the elderly to do) resulting in each individual doing the task they are ‘best’ at^22^. However, within this model, it is assumed that the adults directing care towards the elderly are fecund and therefore physiologically able to increase their fitness directly. This is because, if G3 is fertile, then through investing in G4 she may improve that generations health and longevity, meaning that there is another helping-hand to assist with caring for the younger generations (i.e. G1 in Fig. 1), allowing her to invest energy into direct reproduction. However, as for women the ability to reproduce ends in midlife due to menopause, it means the possible reciprocal benefits to be had from helping elderly parents will wane alongside her fecundity, as after menopause she would no longer be able to translate any reciprocated help into direct reproduction. In line with this, once a woman is post-menopausal and the benefits to be had from investing in older relatives decline (though do not disappear completely), it may be better from a fitness perspective to invest energy into relatives through whom there is a way to increase inclusive fitness, such as children or grandchildren.

Currently, the hypothesis outlining the conditions for elder care to be evolutionarily adaptive has not been tested empirically. As the benefits of elder care are greater if the adult can reproduce, we test the hypothesis using a sample of women from the National Child Development Study (NCDS), some of whom are still menstruating and some of whom who are not, to look at care directed towards parents relative to fecundity status based on the predictions presented in Fig. 1, with this study focussing on the behaviour of G3 from this model. As the benefits of elder care are predicted to decrease if the actor is physiologically post-reproductive, we would expect women who can no longer have children to invest less in their parents than those who can. Further, we additionally test whether there is a difference in downwards intergenerational care relative to fecundity status. If women are optimally allocating their time, then we would predict that women who are no longer menstruating would invest in younger relatives to a greater degree than women who are still fecund. Should caring behaviour relate to fitness outcomes that change relative to fecundity status, it may provide us with some information on how caring norms may have evolved.

## Methods

### Participants

Data were drawn from the NCDS, which is a nationally representative study that has followed a cohort of participants all born in a single week in the United Kingdom since 1958. Since birth, they have been followed up a total of 11 times at ages 7, 11, 16, 23, 33, 42, 44, 46, 50 and 55. As data on time spent caring for grandchildren is only available from the most recent interview, all analyses here are cross-sectional, with all women included in the sample being aged either 55 or 56 (depending on whether the interview was conducted in 2013 or 2014) and representing the third generation of women in Fig. 1. The sample was limited to women who had at least one parent alive and at least one grandchild (n = 934). Data from the NCDS are available from the UK Data Service, and the participant characteristics shown in Supplementary Table S1.

### Variables

#### Hours spent helping parents per week

Information regarding parental caregiving was included as a count variable. In the most recent interviews, participants were asked whether they ever do various activities for their parents (e.g. shopping for them, helping with basic personal needs, giving them lifts, etc.), and if they do, how many hours on average per week do they spend doing so. Any women who reported not helping their parents do any of the activities were coded as helping their parents for zero hours per week.

#### Hours spent caring for grandchildren per month

The number of hours spent caring for grandchildren per month was also included as a count variable. Women were asked whether they ever look after their grandchildren without the grandchild’s parents being present, and if they do, at what frequency and for how many hours. Women who stated that they did not care for their grandchildren or did so less often than monthly were coded as caring for their grandchildren for zero hours per month. This measure also includes overnight stays.

#### Fecundity status at age 55

Fecundity status was derived from information on age, year and reason for last menstrual period, which was collected at ages 44, 50, and 55. Based on this, a binary categorical variable was derived where women were coded as either ‘Still menstruating’ or ‘No longer menstruating’. The latter category comprised of women who were post-menopausal or who had stopped menstruating for another reason, such as a surgical menopause. Women who had stopped menstruating due to menopause or other reasons were grouped together as the direct fitness implications of no longer menstruating are the same, regardless of the reason for it.

#### Control variables

Covariates included were selected based on their expected effect on the woman’s ability to help other family members. As a proxy of socioeconomic status, the age at which the woman left education was included. Employment status was utilised to give an indication of the woman’s time constraints (i.e. if she was employed, it can be expected she had less time to care for kin)^24^, with women being coded as either employed, unemployed, or other, with the latter category including those who are doing something other than formal employment but do not classify themselves as unemployed (e.g. retired, volunteering, studying, etc.). Self-perceived health was used as a measure of how physically able the woman is to help family members^25^, and number of grandchildren was also included to adjust for how many grandparenting responsibilities a woman had. We also included information on the mortality status of the woman’s parents (i.e. whether she had both parents alive or not), which was derived from interviews at ages 7, 11, 16, 23, 42, 46, 50 and 55. The focal woman’s mother’s and father’s age at birth (collected in the perinatal interview) were also included to control for the amount of help her parents may need, as older parents would expected to be more in need of assistance. Finally, in models predicting hours spent caring for parents, time spent caring for grandchildren was adjusted for, and vice versa for models where hours spent caring for grandchildren was the outcome.

### Analyses

Time spent helping parents and caring for grandchildren were both modelled using zero-inflated negative binomial regression (ZINB). This modelling procedure was selected both due to the over-dispersed nature of the data with excess zeros, and because zero-inflated models allow for zeros to be generated through two distinct processes. Here, the model distinguishes between excess zeroes, which occur when the event could not have happened, and true zeros, which occur when there could have been an event. Therefore, the model estimates a binary outcome (does not care versus does care) and a count outcome (the number of hours spent caring). This method is theoretically appropriate, as there are many different reasons people would offer no care to kin: while some people may choose to invest less, for some people the choice is out of their control, with external factors influencing caring behaviours, such as living far away from kin^26^. In addition to this, ZINB was found to better fit the data than negative binomial regression (Supplementary Table S2).

Time spent helping parents was first modelled. A ‘base’ model was first made containing the age the woman left education, employment status, marital status, self-perceived health, number of grandchildren, parent mortality status, age of parents, and time spent caring for grandchildren. Fecundity status was subsequently added, and model fitting then carried out on these two models, utilising their Akaike Information Criterion (AIC) value to understand whether a model including fecundity better fit the data than one without. The model with the lowest AIC value is taken to best fit the data. As AIC values penalise models for complexity, it means the model with the most terms will not automatically be selected as the best. The ΔAIC was also calculated, which is the difference between the candidate models AIC and the AIC value of the best fitting candidate model. If the ΔAIC value is ≤ 2, then it indicates that there is still good evidence to support the candidate model, meaning that a candidate model with a ΔAIC of ≤ 2 is almost as good as the best fitting model. A ΔAIC value of between 4 and 7 is taken to indicate the candidate model has considerably less support, and a ΔAIC of greater than 10 indicates there is no support for the candidate model^27^. The Akaike weights (*w*_i_) were also calculated to evaluate model fit, which give the probability that the candidate model is the best among the set of presented candidate models^27^. The same procedure was then used to model time spent caring for grandchild per month: a model including just the covariates was first made, but this time adjusting for time spent helping parents rather than time caring for grandchildren, with fecundity status then being added, and model fitting was once again carried out using the methods outlined above. All analyses were carried in *R* using the *zeroinfl* function with a negative binomial distribution specified^28^, and model fitting carried out with the package *AICcmodavg*^29^. All visualisations were created using *ggplot2*^30^.

## Results

In this sample of women from the United Kingdom (n = 934), the majority were post-menopausal or had stopped menstruating for another reason (n = 836). Women spent a median of 18 h per month caring for grandchildren (interquartile range [IQR]: 0, 42) and 2 h a week caring for parents (IQR: 0, 5). Time spent caring was slightly greater towards both generations in women who were no longer menstruating, who spent 20 h a month (IQR: 0.00, 48.00) caring for grandchildren and 2 h per week (IQR: 0.00, 5.00) helping parents; compared to 13.5 h a month (IQR: 0, 32) and 1 h a week (IQR: 0, 6) caring for grandchildren and parents, respectively, in women who are still menstruating. Full participant characteristics are shown in Supplementary Table S1.

**Figure 2:**
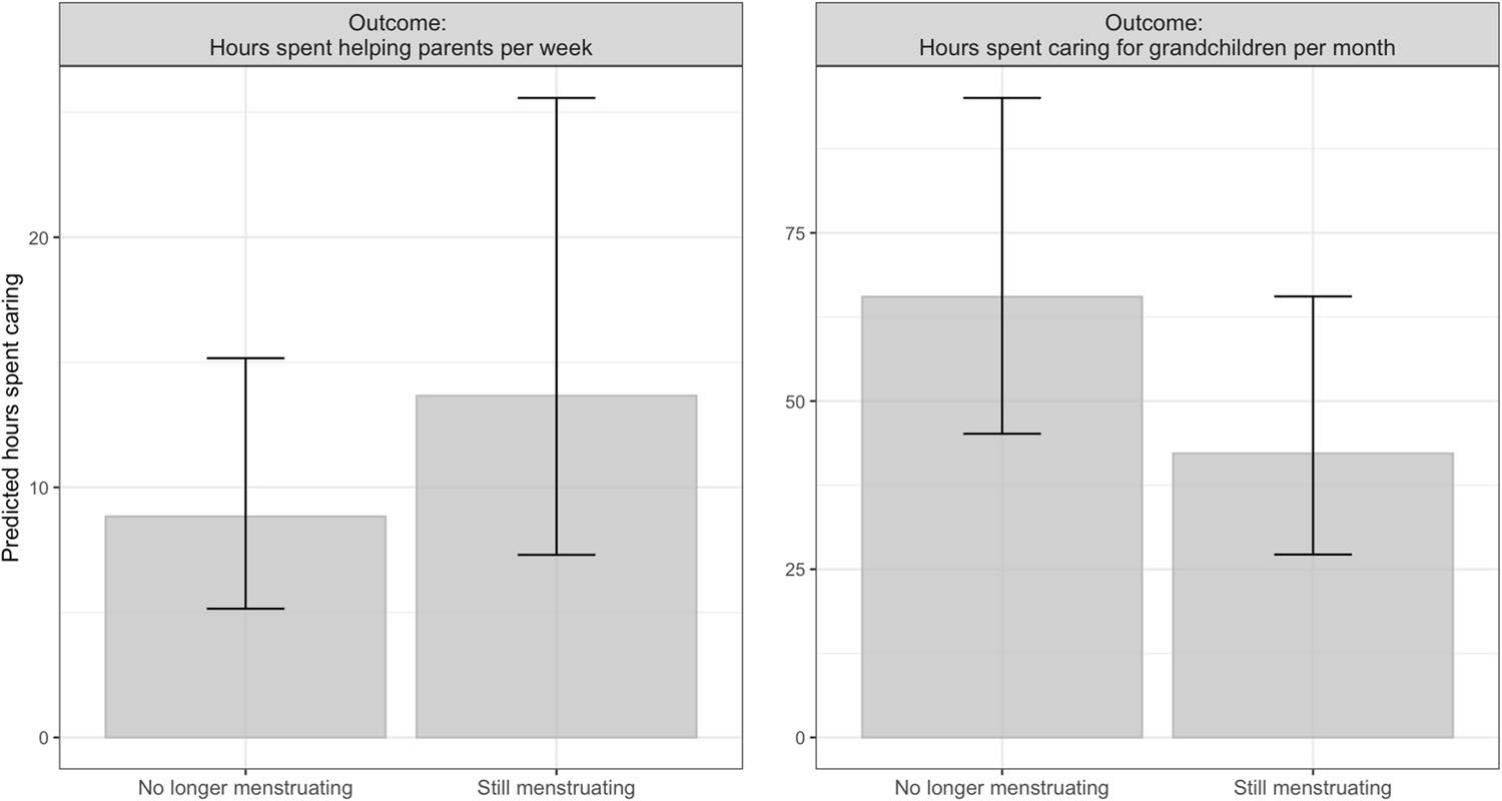
Predicted number of hours spent helping parents and caring for grandchildren relative to fecundity status, based on the results from negative binomial parts of the best fitting models. Error bars indicate the 95% confidence intervals. Models adjust for number of grandchildren, the age the woman left education, employment status, marital status, and health. Where hours spent helping parents is the outcome, time spent caring for grandchildren is also controlled for, and vice versa where hours spent caring for grandchildren is the outcome.

For both outcome variables, the inclusion of fecundity within the models improved their fit to the data based on AIC value (Table 1). Within the negative binomial parts of the best fitting models, women who were no longer menstruating were predicted to spend significantly less time helping parents (incidence rate ratio [IRR]: 0.65, 95% confidence interval [CI]: 0.43–0.97) and more time caring for grandchildren (IRR: 1.55, 95% CI: 1.19–2.02) compared to women who are still menstruating (Fig. 2). The age a woman left education was negatively associated with both caring behaviours, with women who were educated to an older age spending less time caring for both parents (IRR: 0.89, 95% CI: 0.80–1.00) and grandchildren (IRR: 0.94, 95% CI: 0.88–1.01). Being employed predicted significantly less help directed towards parents (IRR: 0.35, 95% CI: 0.19–0.62) but not grandchildren. Variables pertaining to parental health were also related to filial, but not grandchild, care; with having only a father alive (IRR: 2.14, 95% CI: 1.45–3.15) and an older mother (IRR: 1.04, 95% CI: 1.00–1.08) predicting more time spent helping parents. Self-perceived health did not predict either caring behaviour. Within the part of the models predicting excess zeros, no longer menstruating was not a significant predictor of care towards grandchildren (odds ratio [OR]: 0.82, 95% CI: 0.52–1.28) or parents (OR: 0.69, 95% CI: 0.30–1.60). Having just a surviving father was a predictor of parental care (OR: 3.17, 95% CI: 1.56–6.47). Full model results are shown in Supplementary Table S3.

**Table 1:** Results from model fitting based on Akaike Information Criterion (AIC).

Model	K	AIC	ΔAIC	*w*_i_
Outcome = hours spent helping parents				
Covariates	27	4534.02	0.90	0.39
* Fecundity status* + *covariates*	*29*	*4533.12*	*0.00*	*0.61*
Outcome = hours spent caring for grandchildren				
Covariates	27	7184.35	6.59	0.04
* Fecundity status* + *covariates*	*29*	*717,776*	*0.00*	*0.96*

The lowest AIC value is deemed to best fit the data, with ΔAIC referring to the difference in AIC value from the best fitting model (shown in italics). A ΔAIC value of more than two demonstrates a significantly poorer model fit, and *w*_i_ indicates model probability. Covariates include number of grandchildren, age the woman left education, employment status, marital status, and health. Where the outcome is hours spent helping parents, hours spent caring for grandchildren is included in the covariates, and vice versa.

## Discussion

Despite elder care being common cross-culturally, little empirical research from an evolutionary perspective has been conducted looking at the behaviour. We test a hypothesis proposing that upwards intergenerational care will be selected for as long as the actor is able to translate some of the proposed reciprocal benefits of the care into direct fitness, meaning that upwards care being adaptive is somewhat dependent upon the fecundity status of the actor. Based on this, we predicted that women who were still fecund would invest more in elder care than those who were not, as the former would be able to benefit more from any reciprocal care through increasing direct fitness. We further test the assumption that caring behaviour is contingent on the fitness outcomes through looking at whether women who are no longer able to reproduce invest more in younger relatives than women who are still menstruating. In this sample of women from the United Kingdom, support was found for this hypothesis, and it was observed that women who were still menstruating spent more time caring for their parents than women who had stopped (Fig. 2), even after adjusting the potential ‘need’ their parents had for care (e.g. mortality status and parental age). This is in line with the predictions made by Garay et al.^22^: if a fertile woman helps her parents, then they reciprocate the care, therefore allowing her more time to invest in other activities, such as reproduction. As such, the benefits of investing in elderly parents would diminish as women become physiologically post-reproductive, thus explaining the difference in care directed towards parents relative to fecundity status.

We also found that post-reproductive women spent almost double the number of hours per month caring for their grandchildren compared to still menstruating women. This supports existing research suggesting that menopause may have evolved initially due to grandmothers benefits as carers^2,17,18^, and also mimics findings from research by Hofer, et al.^31^ where it was shown that post-menopausal women spend more time grandparenting compared to the control of volunteering. Taken together, these findings suggest that increased levels of care towards younger kin when physiologically post-reproductive might be a behavioural adaptation that was selected for to offset the costs of no longer being able to directly reproduce. This study did not look at whether the grandchildren of women who spend more time with them are actually more successful than grandchildren to whom less time is devoted, as we did not have the data to test this. However, other research has found that investing more in kin does not always translate into increased fitness down the line in modern, post-industrial societies^32^, and that low levels of familial investment in societies with a welfare state (such as the United Kingdom) might be compensated by investment from public goods, such as schooling^33^. We did not find any relationship between fecundity and caring behaviour within the zero-inflated part of the model, but this is likely because caring for kin is often not possible due to restrictions such as proximity^26^, which have no bearing on whether a woman is fecund or not.

It should be noted that the validity of applying this hypothesis could be brought into question based on the age of the women in this data. Our sample included only women aged 55 or 56, and based on reproductive norms and oocyte degradation, even if the women were still menstruating at this age, it is unlikely they would actually reproduce^34,35^. Further to this, the parents of the women in this study are, on average, in their early 80s (Supplementary Table S1), and evidence has shown that the elderly’s benefits as allocarers declines with age^36^, which would suggest that the returns on filial care would also decline. If our tendency to help older generations is a behavioural adaptation that evolved for the reasons proposed by Garay, et al.^22^, it could be that the adaptation is somewhat mismatched to our current environment where there are longer generations and longer lifespans, that have resulted in grandparents being older. However, investing in elderly parents might be reciprocated in ways other than allocare, such as through financial or material rewards, with more filial care possibly resulting in more money or gifts being informally given to the carer or their relatives (e.g. children or grandchildren) as a reciprocal gesture. This form of care was not factored into the original model, however, research has demonstrated that financial transfers from the elderly do not associate with help from the middle generation^37^, meaning that it may not be an incentive. One could also take a more sociological stance and argue that elder care does not require an evolutionary explanation and that it can be explained proximally through various social norms and institutions^38^. However, neither financial rewards nor social norms can explain why there is a significant difference in care-giving behaviour towards parents relative to fecundity status, as we show here.

Being limited to data from only women aged 55 or 56 also meant that few were pre-menopausal. Though the average age of menopause may be increasing^39^, still being fecund at age 55/56 is relatively rare, which is reflected by the fact that only ~ 10% of the women included in this research have not yet stopped menstruating. While there are various reasons women may have stopped menstruating, a proportion would have gone through menopause, and a later menopause is generally associated with being of a higher socioeconomic status and better health^40,41^, and while we have attempted to control for these various lifestyle factors through adjusting for health, employment, and educational attainment, it is likely that the women in this sample who have not yet stopped menstruating are not representative of the ‘average’ middle-aged woman. Ideally, longitudinal data would have been used; but questions regarding grandchild care have only been asked in the 2013/2014 year of interviews meaning it was not possible. It has recently been suggested that longitudinal cohort studies should be utilised in the evolutionary sciences more^42^, however, we are often limited by the variables available.

To our knowledge, this is the first piece of empirical research using real-world data looking at elder care from an evolutionary perspective, in which we have found evidence for kin-directed care by women to be facultatively adjusted up and down the generations based on their own fecundity status.

## Supplementary Information

Supplementary Information.

## Data Availability

The datasets analysed in the current study are available upon application from the UK Data Service.
