# Supplementary material for: An evolutionary perspective on kin care directed up the generations
Source: Sci Rep. 2021 Jul 8;11:14163. doi: 10.1038/s41598-021-93652-4 (PMC8266810; doi:10.1038/s41598-021-93652-4)
Supplement: Supplementary file 1 — Supplementary Information. [file 41598_2021_93652_MOESM1_ESM.pdf]

Supplementary information for

An evolutionary perspective on kin care directed up the generations

Megan Arnot & Ruth Mace

Table S1. Participant characteristics.

	All n (%)/median (IQR)	Still menstruating n (%)/median (IQR)	No longer menstruating n (%)/median (IQR)
n	934	98	836
Hours spent caring for grandchildren per month	18 (0, 48)	13.5 (0, 32)	20 (0, 48)
Hours spent helping parents per week	2 (0, 5)	1 (0, 6)	2 (0, 5)
Fecundity status			
Still menstruating	98 (10.49)	98 (100.00)	0 (0.00)
No longer menstruating	836 (89.51)	0 (0.00)	836 (100.00)
Age left education	16 (16, 17)	16 (16, 18)	16 (16, 17)
Employment status			
Employed	717 (76.77)	80 (81.63)	637 (76.20)
Unemployed	68 (7.28)	6 (6.12)	62 (7.42)
Other	149 (15.95)	12 (12.24)	137 (16.39)
Self-perceived health			
Excellent	101 (10.81)	15 (15.31)	86 (10.29)
Very good/good	615 (65.85)	65 (66.33)	550 (65.79)
Fair	144 (15.42)	11 (11.22)	133 (15.91)

	All n (%)/median (IQR)	Still menstruating n (%)/median (IQR)	No longer menstruating n (%)/median (IQR)
Poor/very poor	74 (7.92)	7 (7.14)	67 (8.01)
Father's age at birth	27 (24, 30)	27 (24, 31.75)	27 (24, 30)
Mother's age at birth	24 (21 27)	24.00 (22, 28)	24.00 (21, 27)
Parent mortality			
Both parents alive	315 (33.73)	40 (40.82)	275 (32.89)
Mother dead, father alive	153 (16.38)	14 (14.29)	139 (16.63)
Mother alive, father dead	466 (49.89)	44 (44.90)	422 (50.48)
Number of grandchildren	2.00 (1, 4)	2 (1, 4)	2 (1, 4)

Table S2. Comparison of models with Vuong's statistic. NB = negative binomial regression, ZINB = zero-inflated negative binomial regression.

Outcome	First model	Second model	Vuong z-statistic	p value	Better model
Parent help	NB	ZINB	-3.623649	<0.001	ZINB
Grandchild care	NB	ZINB	-12.897627	<0.001	ZINB

Table S3. Results from all models using zero-inflated negative binomial regression, reporting incidence rate ratios for the count part of the model and odds ratios for the zero-inflated part, and the 95% confidence intervals.

	Outcome = Hours spent helping parents per week		Outcome = Hours spent caring for grandchildren per month	
	**Covariates**	**Fecundity status + Covariates**	**Covariates**	**Fecundity status + Covariates**
<u>**Count model:**</u>				

	Outcome = Hours spent helping parents per week		Outcome = Hours spent caring for grandchildren per month	
	**Covariates**	**Fecundity status + Covariates**	**Covariates**	**Fecundity status + Covariates**
Fecundity status (ref.: Still menstruating)				
No longer menstruating	-	0.65 (0.43-0.97)	-	1.55 (1.19-2.02)
Age left education	0.91 (0.81-1.01)	0.89 (0.80-1.00)	0.93 (0.87-1.00)	0.94 (0.88-1.01)
Employment status (ref.: Unemployed)				
Employed	0.35 (0.19-0.63)	0.35 (0.19-0.62)	0.83 (0.60-1.15)	0.84 (0.61-1.16)
Other	0.52 (0.28-0.98)	0.49 (0.26-0.91)	1.40 (0.98-2.01)	1.37 (0.96-1.96)
Self-perceived health (ref.: Poor/very poor)				
Fair	1.04 (0.58-1.85)	1.11 (0.63-1.97)	1.09 (0.78-1.53)	1.10 (0.79-1.54)
Very good/good	1.39 (0.80-2.43)	1.42 (0.82-2.46)	1.00 (0.72-1.38)	1.00 (0.72-1.37)
Excellent	0.84 (0.43-1.64)	0.84 (0.44-1.63)	1.03 (0.69-1.53)	1.03 (0.70-1.53)
Parent mortality (ref.: Both parents alive)				
Mother alive, father dead	1.17 (0.90-1.51)	1.21 (0.94-1.57)	1.04 (0.86-1.26)	1.04 (0.86-1.25)
Mother dead, father alive	2.05 (1.39-3.03)	2.14 (1.45-3.15)	0.90 (0.70-1.14)	0.88 (0.69-1.12)

	Outcome = Hours spent helping parents per week		Outcome = Hours spent caring for grandchildren per month	
	**Covariates**	**Fecundity status + Covariates**	**Covariates**	**Fecundity status + Covariates**
Mother's age at birth	1.03 (0.99-1.06)	1.04 (1.00-1.08)	1.00 (0.97-1.02)	0.99 (0.97-1.02)
Father's age at birth	1.02 (0.99-1.05)	1.01 (0.98-1.04)	1.00 (0.98-1.02)	1.00 (0.98-1.02)
Number of grandchildren	1.00 (0.96-1.05)	1.00 (0.95-1.05)	0.99 (0.96-1.02)	0.99 (0.96-1.02)
Hours spent helping parents per week	-	-	1.00 (1.00-1.01)	1.00 (1.00-1.01)
Hours spent caring for grandchildren per month	1.00 (1.00-1.00)	1.00 (1.00-1.00)	-	-
**<u>Zero-inflation model:</u>**				
Fecundity status (ref.: Still menstruating)				
No longer menstruating	-	0.69 (0.30-1.60)	-	0.82 (0.52-1.28)
Age left education	1.09 (0.84-1.42)	1.06 (0.81-1.39)	1.11 (1.00-1.25)	1.11 (0.99-1.24)
Employment status (ref.: Unemployed)				
Employed	0.34 (0.11-1.06)	0.34 (0.11-1.04)	0.94 (0.49-1.78)	0.93 (0.49-1.77)
Other	0.50 (0.14-1.78)	0.48 (0.14-1.66)	1.09 (0.55-2.17)	1.09 (0.55-2.17)
Self-perceived health (ref.: Poor/very poor)				

	Outcome = Hours spent helping parents per week		Outcome = Hours spent caring for grandchildren per month	
	Covariates	Fecundity status + Covariates	Covariates	Fecundity status + Covariates
Fair	0.57 (0.14-2.24)	0.66 (0.18-2.39)	0.61 (0.32-1.17)	0.61 (0.32-1.18)
Very good/good	0.81 (0.26-2.51)	0.82 (0.27-2.51)	0.81 (0.44-1.50)	0.82 (0.45-1.50)
Excellent	0.69 (0.14-3.44)	0.69 (0.14-3.37)	1.11 (0.55-2.25)	1.11 (0.55-2.24)
Parent mortality (ref.: Both parents alive)				
Mother alive, father dead	0.45 (0.17-1.17)	0.47 (0.18-1.18)	0.99 (0.71-1.37)	0.99 (0.71-1.38)
Mother dead, father alive	3.10 (1.52-6.33)	3.17 (1.56-6.47)	1.05 (0.69-1.61)	1.06 (0.69-1.62)
Mother’s age at birth	0.93 (0.83-1.03)	0.94 (0.84-1.04)	1.03 (0.99-1.08)	1.03 (0.99-1.08)
Father’s age at birth	0.97 (0.87-1.08)	0.96 (0.86-1.07)	0.99 (0.95-1.03)	0.99 (0.95-1.03)
Number of grandchildren	0.97 (0.86-1.09)	0.96 (0.85-1.09)	0.94 (0.88-1.00)	0.94 (0.89-1.00)
Hours spent helping parents per week	-	-	0.94 (0.91-0.97)	0.94 (0.91-0.97)
Hours spent caring for grandchildren per month)	0.99 (0.97-1.00)	0.99 (0.97-1.00)	-	-
